# Postnatal β2 adrenergic treatment improves insulin sensitivity in lambs with IUGR but not persistent defects in pancreatic islets or skeletal muscle

**DOI:** 10.1113/JP278726

**Published:** 2019-11-29

**Authors:** Dustin T. Yates, Leticia E. Camacho, Amy C. Kelly, Leah V. Steyn, Melissa A. Davis, Andrew T. Antolic, Miranda J. Anderson, Ravi Goyal, Ronald E. Allen, Klearchos K. Papas, William W. Hay, Sean W. Limesand

**Affiliations:** ^1^ School of Animal and Comparative Biomedical Sciences University of Arizona Tucson AZ USA

**Keywords:** cardiovascular dysfunction, developmental origins of health and disease, fetal programming, glucose disposal rates, metabolic syndrome

## Abstract

**Key points:**

- Previous studies in fetuses with intrauterine growth restriction (IUGR) have shown that adrenergic dysregulation was associated with low insulin concentrations and greater insulin sensitivity.
- Although whole‐body glucose clearance is normal, 1‐month‐old lambs with IUGR at birth have higher rates of hindlimb glucose uptake, which may compensate for myocyte deficiencies in glucose oxidation.
- Impaired glucose‐stimulated insulin secretion in IUGR lambs is due to lower intra‐islet insulin availability and not from glucose sensing.
- We investigated adrenergic receptor (ADR) β2 desensitization by administering oral ADRβ modifiers for the first month after birth to activate ADRβ2 and antagonize ADRβ1/3. In IUGR lambs ADRβ2 activation increased whole‐body glucose utilization rates and insulin sensitivity but had no effect on isolated islet or myocyte deficiencies.
- IUGR establishes risk for developing diabetes. In IUGR lambs we identified disparities in key aspects of glucose‐stimulated insulin secretion and insulin‐stimulated glucose oxidation, providing new insights into potential mechanisms for this risk.

**Abstract:**

Placental insufficiency causes intrauterine growth restriction (IUGR) and disturbances in glucose homeostasis with associated β adrenergic receptor (ADRβ) desensitization. Our objectives were to measure insulin‐sensitive glucose metabolism in neonatal lambs with IUGR and to determine whether daily treatment with ADRβ2 agonist and ADRβ1/β3 antagonists for 1 month normalizes their glucose metabolism. Growth, glucose‐stimulated insulin secretion (GSIS) and glucose utilization rates (GURs) were measured in control lambs, IUGR lambs and IUGR lambs treated with adrenergic receptor modifiers: clenbuterol atenolol and SR59230A (IUGR‐AR). In IUGR lambs, islet insulin content and GSIS were less than in controls; however, insulin sensitivity and whole‐body GUR were not different from controls. Of importance, ADRβ2 stimulation with β1/β3 inhibition increases both insulin sensitivity and whole‐body glucose utilization in IUGR lambs. In IUGR and IUGR‐AR lambs, hindlimb GURs were greater but fractional glucose oxidation rates and *ex vivo* skeletal muscle glucose oxidation rates were lower than controls. Glucose transporter 4 (GLUT4) was lower in IUGR and IUGR‐AR skeletal muscle than in controls but GLUT1 was greater in IUGR‐AR. ADRβ2, insulin receptor, glycogen content and citrate synthase activity were similar among groups. In IUGR and IUGR‐AR lambs heart rates were greater, which was independent of cardiac ADRβ1 activation. We conclude that targeted ADRβ2 stimulation improved whole‐body insulin sensitivity but minimally affected defects in GSIS and skeletal muscle glucose oxidation. We show that risk factors for developing diabetes are independent of postnatal catch‐up growth in IUGR lambs as early as 1 month of age and are inherent to the islets and myocytes.

## Key points

- Previous studies in fetuses with intrauterine growth restriction (IUGR) have shown that adrenergic dysregulation was associated with low insulin concentrations and greater insulin sensitivity.
- Although whole‐body glucose clearance is normal, 1‐month‐old lambs with IUGR at birth have higher rates of hindlimb glucose uptake, which may compensate for myocyte deficiencies in glucose oxidation.
- Impaired glucose‐stimulated insulin secretion in IUGR lambs is due to lower intra‐islet insulin availability and not from glucose sensing.
- We investigated adrenergic receptor (ADR) β2 desensitization by administering oral ADRβ modifiers for the first month after birth to activate ADRβ2 and antagonize ADRβ1/3. In IUGR lambs ADRβ2 activation increased whole‐body glucose utilization rates and insulin sensitivity but had no effect on isolated islet or myocyte deficiencies.
- IUGR establishes risk for developing diabetes. In IUGR lambs we identified disparities in key aspects of glucose‐stimulated insulin secretion and insulin‐stimulated glucose oxidation, providing new insights into potential mechanisms for this risk.

## Introduction

Intrauterine growth restriction (IUGR) and early catch‐up growth predict later development of chronic non‐communicable metabolic disorders such as Type 2 diabetes and cardiovascular disease (Barker, 1990, 1993, 2002; Barker *et al*. 1993a, 2005, 2007; Soto *et al*. 2003; McMillen & Robinson, 2005; Whincup *et al*. 2008). Deficiencies in insulin secretion and insulin sensitivity manifest early in children that were born small‐for‐gestational‐age (SGA) and eventually progress into more pronounced metabolic pathologies that characterize the metabolic syndrome (Barker *et al*. 1993b, 2002; Hofman *et al*. 1997; Li *et al*. 2001; Bazaes *et al*. 2004; McMillen *et al*. 2006, 2008; Eriksson, 2019). Recapitulating the causal link between fetal growth restriction and lifelong metabolic disorders in numerous experimental models across a variety of species has implicated adaptive developmental programming in response to placental insufficiency as the underlying mechanism (Simmons *et al*. 2001; Ford *et al*. 2007; Owens *et al*. 2007b; Jimenez‐Chillaron *et al*. 2009; Gatford *et al*. 2010; Wallace *et al*. 2018; Chatmethakul & Roghair, 2019).

Fetuses with placental insufficiency experience progressive nutrient and oxygen deprivation and respond with greater noradrenaline (norepinephrine) and adrenaline (epinephrine) secretion (Greenough *et al*. 1990; Simonetta *et al*. 1997; Danielson *et al*. 2005; Limesand & Rozance, 2017). We found that when placental insufficiency was produced by maternal hyperthermia in sheep, the resulting fetal hypercatecholaminaemia inhibited insulin secretion and independently slowed growth (Leos *et al*. 2010; Limesand *et al*. 2013; Macko *et al*. 2013, 2016; Davis *et al*. 2015). Throughout life, individuals born SGA due to IUGR have less lean mass and reduced muscle strength, which is worsened by slower muscle accretion during infancy (Greenwood *et al*. 1998; Hediger *et al*. 1998; Gale *et al*. 2001; Sayer *et al*. 2004; Kensara *et al*. 2005; Inskip *et al*. 2007; Yliharsila *et al*. 2007). Despite less muscle mass, insulin sensitivity for glucose utilization is greater in IUGR fetal sheep, as their whole‐body net glucose utilization rates are normal but plasma insulin and glucose concentrations are lower (Limesand *et al*. 2007; Thorn *et al*. 2013). However, the fraction of glucose utilized for oxidative metabolism is lower, which complements previous findings that chronic adrenergic receptor β (ADRβ) stimulation increases non‐oxidative glucose disposal (Scheidegger *et al*. 1984; Budohoski *et al*. 1987; Jensen *et al*. 2005; Limesand *et al*. 2007; Brown *et al*. 2015). We found that greater insulin sensitivity for glucose utilization persists in IUGR lambs at 2 weeks of age, but their higher plasma lactate concentrations may indicate that rates of glucose oxidation do not recover, resulting in lactate production; these results are similar to observations in children born SGA from IUGR (Jornayvaz *et al*. 2004; Camacho *et al*. 2017). Furthermore, glucose‐stimulated insulin secretion (GSIS) was substantially greater in these IUGR lambs, which we associated with compensatory adaptations to high catecholamines *in utero* (Leos *et al*. 2010; Chen *et al*. 2014, 2017; Camacho *et al*. 2017; Kelly *et al*. 2018). These findings indicate a role for changes in ADRβ signalling that creates a postnatal enhancement of GSIS and insulin‐stimulated glucose utilization (Limesand & Rozance, 2017; Yates *et al*. 2018).

The three ADRβ isoforms are expressed in a tissue‐specific manner, and as G‐protein coupled receptors, their persistent activation lowers their own responsiveness (Collins *et al*. 1991; Wettschureck & Offermanns, 2005). In near‐term IUGR fetuses and in 3‐week‐old IUGR lambs, we have shown that expression of ADRβ2 mRNA is downregulated in adipose tissue and skeletal muscle (Chen *et al*. 2010; Yates *et al*. 2012a). This ADRβ2 deficiency could explain restricted growth and muscle accretion in IUGR offspring because stimulation with ADRβ2 agonists repartitions nutrients in growing animals to support anabolic protein accretion and energy‐producing oxidative pathways (Byrem *et al*. 1998; Consolo *et al*. 2015; Cadaret *et al*. 2017). Furthermore, deficiencies in glucose oxidation in IUGR fetal sheep and in adult humans born SGA from IUGR coincide with impaired proximal insulin signalling that may reflect the loss of counter‐regulatory effects of adrenergic signalling (Hadcock *et al*. 1992; Morisco *et al*. 2005; Ozanne *et al*. 2005; Thorn *et al*. 2009). An interaction in signal transduction pathways has been demonstrated for ADRβ2 and insulin receptors, and lower ADRβ2 concentrations relative to ADRβ1 would favour inhibition of proximal insulin signalling (Baltensperger *et al*. 1996; Wang *et al*. 2000; Castle *et al*. 2001; Brennesvik *et al*. 2005; Gavi *et al*. 2007). Because this ADRβ profile is still observed in young lambs with IUGR at birth, we hypothesize that the ADRβ2 effects to inhibit insulin receptors and insulin signalling continue postnatally even though insulin secretion is no longer suppressed (Muhlhausler *et al*. 2009; Chen *et al*. 2010; Camacho *et al*. 2017).

Although multiple factors may contribute to glucose intolerance, we postulate that selective desensitization of ADRβ2 after persistent exposure to hypercatecholaminaemia in late gestation represents an adaptive mechanism responsible for impaired skeletal muscle growth and insulin‐sensitive glucose metabolism. Furthermore, previous studies of the fetal cardiovascular system indicate that sustained high concentrations of catecholamines lower ADRβ responsiveness in the heart (Jones & Ritchie, 1978; Bassett *et al*. 1990; Bocking *et al*. 1995; Gardner *et al*. 2002). Therefore, we investigated whether selective ADRβ1 and ADRβ3 antagonists combined with an ADRβ2 agonist will normalize the predicted adrenergic dysregulation and improve early outcomes in growth, insulin secretion and action, and cardiovascular parameters in young lambs that were IUGR at birth.

Our objectives for this study were to determine deficiencies in insulin‐stimulated glucose metabolism in 1‐month‐old lambs with IUGR at birth. We tested the hypothesis that these lambs have impaired GSIS and greater hindlimb glucose utilization rates. Furthermore, we postulate that these limitations result from intrinsic impairments for GSIS responsiveness of islets and for skeletal muscle insulin‐stimulated glucose metabolism. Finally, we sought to determine whether ADRβ2 activation via daily administration of pharmaceutical ADRβ modifiers would increase insulin sensitivity for glucose metabolism and growth in IUGR lambs.

## Methods

### Ethical approval

Study protocols were approved by the Institutional Animal Care and Use Committee at The University of Arizona (Protocol no. 08‐132) and follow the guidelines from the American Association for the Accreditation of Laboratory Animal Care International. All work was conducted at the university's Agricultural Research Centre. Pregnant Columbia–Rambouillet crossbred ewes were purchased from Nebeker Ranch (Lancaster, CA, USA), and those carrying singleton pregnancies were identified by ultrasonography prior to being assigned to an experimental group. The ewes were 2–4 years of age with unknown parity. Animals were managed as previously described (Chen *et al*. 2010), and these pregnant ewes (45 ± 2 kg) were assigned via simple randomization to the thermoneutral control group (*n* = 16) or the placental insufficiency‐induced IUGR group (*n* = 30). Lambs born from the placental insufficiency‐induced IUGR group (IUGR lambs) were produced using the maternal hyperthermia model (Chen *et al*. 2010; Camacho *et al*. 2017). Briefly, pregnant ewes were exposed to elevated ambient temperatures (40°C for 12 h; 35°C for 12 h; dew point 22°C) from 38 ± 1 to 87 ± 1 days of gestation. Control lambs were from ewes that were maintained at 22 ± 1°C and pair fed to the average *ad libitum* feed intake of the hyperthermic ewe group. All sheep were given *ad libitum* access to water and salt. One control and nine IUGR fetuses were lost prior to birth for undiagnosed reasons, leaving 15 controls and 22 IUGR lambs to be studied. Ewes delivered naturally and lambs were removed from the ewe to eliminate confounding maternal variability. Lambs were ear tagged and housed in adjacent individual pens in a separate location from their mothers. All lambs were fed colostrum four to six times over the first 36 h after birth before being reared solely on *ad libitum* milk replacer (Milk Specialties Co., Dundee, IL, USA). Body weight, crown–rump length (poll to tail head), hindlimb length (hip to hoof) and head circumference were measured at birth. The first seven IUGR lambs and six control lambs born were pre‐selected for postnatal studies without being subjected to the postnatal intervention. The remaining IUGR lambs were randomly assigned to also receive either no postnatal intervention (IUGR; *n* = 14) or to receive daily oral ADRβ modifiers (IUGR‐AR, *n* = 8). This daily treatment consisted of 20 µg kg^−1^ day^−1^ clenbuterol (ADRβ2 agonist), 400 µg kg^−1^ day^−1^ atenolol (ADRβ1 antagonist) and 2 µg kg^−1^ day^−1^ SR59230A (ADRβ3 antagonist), given orally in 50 ml of milk replacer (Coleman *et al*. 1988; MacRae *et al*. 1988; Manara *et al*. 1996; Torneke *et al*. 1998; Chiou *et al*. 2000; Despres *et al*. 2002; Kanzler *et al*. 2011; Miniaci *et al*. 2013). The doses of clenbuterol, atenolol and SR59230A were chosen to provide the lowest effective dose in order to minimize potential off‐target effects. Because of the prospective nature of the study and application of hyperthermia before the ability to determine fetal sex, the study did not take into account the sex distribution.

Physiological studies to measure insulin secretion and insulin sensitivity were performed on each lamb in no particular order and separated by at least 1 day. Growth rates were determined from body weights measured daily from birth (day 0) to day 29 of age. Absolute growth rates were linear over this period, and the *R* ^2^ (coefficient of determination) values of all slopes were ≥0.96 and not different among experimental groups. Growth rates as a percentage of birth weights were calculated by subtracting birth weight from daily body weight and then dividing by birth weight. Dry matter intake per gram of weight gained was used to determine daily feed‐to‐gain efficiency for each lamb (i.e. grams of milk consumed on a dry matter basis per gram of weight gained).

### Surgical preparation

At 24 ± 1 days of age, lambs (15 control, 14 IUGR and 8 IUGR‐AR) were fasted for 3–4 h. A jugular vein was used to administer diazepam (0.2 mg kg^−1^) and ketamine (20 mg kg^−1^) for induction, and the lambs were intubated and maintained by inhalation of 1.5–4% isoflurane in oxygen for the duration of the surgical procedure. The depth of anaesthesia was determined and maintained by response to touch, corneal reflex and assessment of muscle tone, as well as continuous pulse oximetry and heart rate monitoring. At induction, lambs received an intramuscular injection of penicillin G procaine injectable suspension (1350 units (U) kg^−1^; Agri‐Cillin, Huvepharma, Inc., Peachtree City, GA, USA). In the non‐study hindlimb, indwelling catheters (Tygon ND‐100‐80 Flexible Plastic Tubing; outer diameter 1.4 mm and inner diameter 0.9 mm) were surgically placed in the descending (abdominal) aorta and inferior vena cava via the femoral artery and vein for blood sampling and intravenous infusions. In the distal femoral vein of the contralateral hindlimb, a catheter was placed with the tip advanced to the external iliac vein and the deep circumflex iliac artery and vein were ligated and severed to isolate blood flow to the external iliac artery and vein. This hindlimb was designated the study limb. A Precision S‐series Flow Probe (3 or 4 mm; Transonic Systems, Inc., Ithaca, NY, USA) was positioned around the external iliac artery of the study limb. Prior to placement, catheters were filled with heparinized saline (30 U ml^−1^, 0.9% w/v NaCl, Nova‐Tech, Inc., Grand Island, NE, USA). After placement, the flow probe cable and the catheters were tunnelled subcutaneously to the flank, exteriorized through a skin incision, and kept in a plastic mesh pouch sutured to the skin. Lambs were given post‐operative analgesics (0.01 g kg^−1^ body weight phenylbutazone) for 3 days and allowed to recover before performing studies. Catheters were flushed daily with heparinized saline.

### Insulin secretion responsiveness to glucose and arginine

Glucose‐stimulated insulin secretion (GSIS) was evaluated with a square‐wave hyperglycaemic clamp at 28 ± 1 days of age as described previously (Camacho *et al*. 2017). Briefly, lambs were fasted for 3 h and then placed into the Panepinto sling. After approximately 20 min of acclimation, basal (fasted) blood samples were collected at −30, −11 and −2 min for plasma glucose, insulin, cortisol, adrenaline and noradrenaline measurements. The hyperglycaemic clamp was initiated with an intravenous dextrose bolus (250 ± 20 mg kg^−1^) followed by a constant infusion of 33% (w/v) dextrose solution that was adjusted to maintain arterial plasma glucose concentrations at approximately twice the basal glucose concentration observed for each lamb. Sample times are presented relative to administration of the dextrose bolus (time = 0). After the onset of the infusion, arterial blood samples were collected every 5 min for a minimum of 20 min to ensure that steady‐state plasma glucose concentrations were achieved. Afterward, three blood samples were collected at approximately 30, 45 and 60 min. Steady‐state hyperglycaemic conditions were considered to be confirmed when glucose concentrations varied less than ±9% from the overall mean. Acute, first‐phase insulin concentrations were calculated for the first 20 min of hyperglycaemia and second‐phase insulin concentrations were calculated during the hyperglycaemic clamp (30–60 min). Glucose‐potentiated arginine‐stimulated insulin concentration was determined with a follow‐on arginine bolus (0.5 mmol kg^−1^) to the GSIS study. One minute after the final GSIS sample was collected, the dose of arginine was administered over a 4 min period. Blood samples for plasma insulin concentrations were collect at 5 and 15 min after administering arginine.

### Insulin sensitivity for glucose disposal rate

At 28 ± 1 days of age, glucose utilization rates were measured under basal (fasted) conditions and then again during a hyperinsulinaemic–euglycaemic clamp as described previously (Camacho *et al*. 2017). Body weight‐specific rates of glucose utilization were determined by the net disappearance rate of d‐[^14^C‐U]glucose (PerkinElmer Life Sciences, Boston, MA, USA) during basal and hyperinsulinaemic steady‐state periods. Lambs were fasted for 3 h and then placed into the Panepinto sling. A constant infusion (2 ml h^−1^) of radiolabelled glucose (37.2 µCi ml^−1^) in saline was initiated following a 4 ml priming bolus. After 40 min, four arterial blood samples were collected at 8–10 min intervals and used to measure basal glucose utilization rates (µmol min^−1^ kg^−1^), plasma insulin, glucose and lactate concentrations, and arterial blood gases and oximetry. Hyperinsulinaemia was initiated with a priming dose of insulin (175 mU kg^−1^; HumulinR; Lilly; Indianapolis, IN, USA) followed by a constant infusion at 0.5, 2 or 4 mU min^−1^ kg^−1^. Each lamb was studied at a minimum of two hyperinsulinaemic periods. Euglycaemia was concurrently maintained with a 33% (w/v) dextrose infusion that was adjusted in response to arterial plasma glucose concentrations measured every 5–10 min until steady‐state conditions were achieved, usually within an hour. Euglycaemia was considered to be at a steady state when arterial plasma glucose concentrations varied less than ±9% of the basal period mean. Arterial blood samples were collected at 8–10 min intervals.

For lambs in which hindlimb venous catheters remained patent on the day of the study (*n* = 7 controls, 5 IUGR, 5 IUGR‐AR), hindlimb glucose uptake and oxidation rates were determined. Blood flow into the hindlimb through the exterior iliac artery was measured with the Transonic flow probe and recorded using LabChart software (ADInstruments, Colorado Springs, CO, USA). Venous blood samples from the study limb were collected simultaneously with arterial blood samples. Aliquots of arterial and venous whole blood samples were used to determine blood [^14^C]glucose concentrations, blood ^14^CO_2_ concentrations, blood oxygen content, and plasma glucose and lactate concentrations. Hindlimb glucose oxidation rates were measured at 0 mU min^−1^ kg^−1^ (basal) and 4 mU min^−1^ kg^−1^ hyperinsulinaemic–euglycaemic periods.

### Heart rate and blood pressure

To confirm the functional presence of orally administered β2 agonist clenbuterol along with β1 antagonist atenolol and β3 antagonist SR59230A we measured mean systemic arterial blood pressures and heart rates by attaching an externalized arterial catheter to a physiological pressure transducer that was connected to a PowerLab 8/35 with a bridge amplifier (ADInstruments Inc.). Pressures and heart rates were determined on three separate days and analysed with LabChart software. Pressure transducers were calibrated with a mercury column manometer. Lambs were placed in the sling and physiological pressure transducers were set to the height of the heart. After an acclimation period of at least 10 min, data were recorded for a minimum of eight consecutive minutes under basal conditions. At 29 ± 1 days of age, lambs were challenged with the ADRβ1 agonist dobutamine HCl (12.5 mg ml^−1^; Hospira, Inc., Lake Forest, IL, USA) to evaluate cardiac responsiveness (Stephens *et al*. 2011). Data were recorded for 15 min during both basal and dobutamine‐stimulated (10 µg min^−1^ kg^−1^) periods.

### Biochemical analysis and calculations

Plasma glucose and lactate concentrations were measured with a YSI 2700 Select Biochemistry Analyzer (Yellow Springs Instruments, Yellow Springs, OH, USA). Blood gases and oximetry were measured in whole blood collected in heparin‐lined syringes (Elkins‐Sinn, Cherry Hill, NJ, USA) using an ABL720 (Radiometer, Copenhagen, Denmark). Values were temperature‐corrected for the rectal temperature of the lamb measured at the start of the study. Whole blood [^14^C]glucose was determined in supernatants after being deproteinized by mixing whole blood with 0.3 N zinc sulfate heptahydrate and 0.3 M barium hydroxide. The supernatant was separated into triplicate aliquots and dried. Radioactivity was measured with a LS 6500 Multi‐Purpose Scintillation Counter (Beckman Coulter, Fullerton, CA, USA) in Hionic Fluor Liquid Scintillation Cocktail (PerkinElmer Inc., Waltham, MA, USA). Plasma hormone concentrations were determined by enzyme‐linked immunosorbent assay (ELISA) for insulin (Ovine Insulin ELISA; ALPCO Diagnostics, Windham, NH, USA; sensitivity 0.14 ng ml^−1^ intra‐ and interassay coefficients of variation, 3% and 6%, respectively), cortisol (Oxford Biomedical Research, Oxford, MI, USA; sensitivity 10 pg ml^−1^; intra‐ and interassay coefficients of variation, 9% and 12%, respectively), noradrenaline (Labor Diagnostika Nord, Nordhorn, Germany; sensitivity, 25 pg ml^−1^; intra‐ and interassay coefficients of variation, 6% and 14%, respectively) and adrenaline (Labor Diagnostika Nord; sensitivity, 8.3 pg ml^−1^; intra‐ and interassay coefficients of variation, 11% and 17%, respectively). The ^14^CO_2_ was released from triplicate aliquots of whole blood with 2 N HCl, captured in Solvable (PerkinElmer Inc.), and measured in Ultima Gold scintillation cocktail (PerkinElmer Inc.).

Whole‐body net glucose utilization rates (µmol min^−1^) were calculated as the ratio of [^14^C]glucose infusion rate (d.p.m. min^−1^) to arterial whole blood [^14^C]glucose specific activity (d.p.m. µmol^−1^ glucose). Endogenous (hepatic) glucose production rates were calculated as the difference between the whole‐body net glucose utilization rate and exogenous dextrose (d‐glucose) infusion rate (µmol min^−1^). All rates were normalized to body weight (kg). Insulin sensitivity for glucose utilization rate (µmol min^−1^ kg^−1^ µg^−1^ l^−1^) was calculated as the body weight‐specific net glucose utilization rate (µmol min^−1^ kg^−1^) divided by the arterial plasma insulin concentration (µg l^−1^).

### Hindlimb metabolic flux calculation

Mean hindlimb glucose, lactate, [^14^C]glucose and ^14^CO_2_ fluxes were calculated from the simultaneously collected femoral arterial and venous blood sample pairs. Weight‐specific net uptake rates of oxygen, [^14^C]glucose and glucose as well as outputs of lactate were calculated by the Fick principle as the product of artery blood flow and arteriovenous difference (Rozance *et al*. 2018). Arterial blood [^14^C]glucose concentrations (d.p.m. ml^−1^) were divided by arterial blood glucose concentrations (µmol ml^−1^) to determine specific activity (d.p.m. µmol^−1^). All net hindlimb uptakes and output rates were normalized to hindlimb weight (kg) measured at necropsy. Glucose and lactate oxygen quotients were calculated as six or three times, respectively, the ratio of the arteriovenous difference of the respective carbohydrate to the arteriovenous difference of oxygen. The fractional extraction (%) of glucose across the hindlimb was calculated as the arteriovenous difference in plasma glucose concentration divided by the arterial plasma glucose concentration.

### Post‐mortem

Lambs were killed at 31 ± 1 days with an intravenous overdose of sodium pentobarbital (86 mg kg^−1^) and phenytoin sodium (11 mg kg^−1^; Euthasol; Virbac Animal Health). At necropsy, pancreatic ducts were perfused with a collagenase solution (0.5 mg ml^−1^ Collagenase V, Sigma‐Aldrich, St Louis, MO, USA; 0.02% DNase I, Roche, Indianapolis, IN, USA; in Krebs–Ringer buffer (KRB), 118 mmol l^−1^ NaCl, 4.8 mmol l^−1^ KCl, 25 mmol l^−1^ NaHCO_3_, 1.2 mmol l^−1^ MgSO_4_, 1.2 mmol l^−1^ KH_2_PO_4_, 2.5 mmol l^−1^ CaCl_2_) as described previously (Limesand *et al*. 2006). The pancreas was removed by blunt dissection, submerged in the collagenase solution, and incubated at 37°C for 20 min with gentle mixing every 3 min for islet isolation. Organs (brain, liver, heart, kidneys and lungs) and perirenal adipose tissue were dissected and weighed. Mid‐sections of semitendinosus, semimembranosus and bicep femoris muscles were collected for cell isolation or were frozen. The study limb was disarticulated at the proximal end of the femur and weighed. Tissue samples were snap‐frozen in liquid nitrogen and stored at −80°C for enzyme and immunoblot analysis.

### Pancreatic islet isolation and functional assessments

Digested pancreas tissue was filtered through a 500 µm mesh filter and was washed three times by sedimentation in KRB containing 0.5% bovine serum albumin (BSA). Pancreas tissue that did not pass through the mesh filter was subjected to an additional 20 min digestion in collagenase solution and then filtered and washed. Islets were partially purified with a discontinuous gradient of polysucrose 400 (Corning cellgro, Corning, NY, USA) diluted with Hank's balanced salt solution (Gibco HBSS; Thermo Fisher Scientific, Waltham, MA, USA) to 25%, 23%, 20% and 11% dilutions. After being centrifuged at 1400 *g* for 20 min, islets were removed from the 20% layer and were washed three times in KRB–BSA. Islets were incubated overnight in RPMI 1640 (Sigma‐Aldrich) supplemented with 5% fetal bovine serum and penicillin–streptomycin–neomycin (0.1 mg ml^−1^ − 0.1 mg ml^−1^–0.2 mg ml^−1^) at 37°C in 95% O_2_ − 5% CO_2_.

Islets were incubated for 60 min in KRB–BSA prior to functional assessments. Islets (∼200 per lamb) from each lamb were re‐suspended in Media 199 (Corning Mediatech, Inc., Tewksbury, MA, USA) that had been pre‐warmed to 37°C and were divided evenly between three chambers of a Fluorescence Lifetime Micro Oxygen Monitoring System (Instech Laboratories, Inc., Plymouth Meeting, PA, USA) (Papas *et al*. 2007; Smith *et al*. 2017). Measurements of partial pressure of O_2_ ($P_{O_{2}}$) in each chamber were recorded over time using fibre optic sensors and NeoFox viewer software (Instech Laboratories, Inc.). Oxygen consumption rates (OCRs; nmol O_2_ min^−1^) were determined from the slope of $P_{O_{2}}$ disappearance over time and normalized to the DNA content of islets in each chamber (OCR/DNA; nmol O_2_ min^−1^ (mg DNA)^−1^). Islet DNA was extracted with a 1 N ammonium hydroxide and 0.2% Triton X‐100 solution and DNA content was determined in triplicate with Quant‐iT PicoGreen dsDNA kit (Thermo Fisher Scientific) according to manufacturer instructions. Oxygen consumption rates were measured in control and IUGR islets only.

Insulin secretion from isolated islets was measured by perifusion (Biorep Technologies Perifusion System, Peri‐4.2; Miami Lakes, FL, USA). GSIS was measured in triplicate with 75 islets per perifusion chamber at a flow rate of 100 µl min^−1^. KRB–BSA that was supplemented with glucose (0.5 or 11.1 mmol l^−1^) or KCl (30 mmol l^−1^ with 1.1 mmol l^−1^ glucose), pre‐warmed to 37°C, and oxygen‐saturated (95% O_2_–5% CO_2_) was used in islet perifusions. Following a 40 min baseline period at 0.5 mM glucose, islets were stimulated for 40 min with 11.1 mmol l^−1^ glucose (GSIS) and subsequently with KCl–glucose (maximal response). Samples were collected and stored at −80°C, and insulin concentrations were subsequently measured with an ovine insulin ELISA. First phase insulin secretion was determined over the first 11 min of high glucose and second phase insulin secretion was determined between 20 and 40 min by calculating the area under the curve for these time frames. Islet preparations that were unresponsive to KCl stimulation were excluded from the analysis. Islet insulin contents were determined in five replicates of 10 islets as described previously (Limesand *et al*. 2006).

### *Ex vivo* skeletal muscle glucose oxidation rates

Longitudinal strips of semitendinosus muscle (6 technical replicates per condition for each lamb) were isolated and glucose oxidation rates were determined as described previously (Cadaret *et al*. 2017) with some modifications. Muscle strips (30–50 mg) were dissected, mounted in Plexiglas U‐clamps, and placed in 6‐well plates (Costar, Corning Inc., Kennebunk, ME, USA). Muscle strips were pre‐incubated for 1 h at 37°C (95% O_2_: 5% CO_2_) in oxygen‐saturated Krebs–Henseleit bicarbonate buffer (KHB, pH 7.4) supplemented with 0.1% BSA, 5 mmol l^−1^ d‐glucose and 32 mmol l d‐mannitol (Sigma‐Aldrich). Muscle strips were then incubated in KHB media containing no added hormones (basal), insulin (10 µU ml^−1^ Humulin R), insulin + catecholamines (12.5 µmol l^−1^ adrenaline,12.5 µmol l^−1^ noradrenaline), or insulin + cytochalasin B (20 mmol l^−1^; Sigma‐Aldrich) for 30 min. Finally, muscle strips were incubated for 1 h in the above treatment media supplemented with d‐[^14^C‐U]glucose (2 µCi ml^−1^). Glass microfibre filters (Whatman GF/D; GE Healthcare Life Sciences, Little Chalfont, UK) were saturated with 1 M NaOH and suspended over each well of the plate, which was sealed with a plastic gasket. After 1 h, 1 N HCl was injected into each well. Plates were incubated for 2 h at room temperature, the filter papers were removed and radioactivity from captured ^14^CO_2_ was measured via liquid scintillation in Biosafe II Scintillation cocktail (Research Products International Corp., Mount Prospect, IL, USA). Muscle strips were removed from clamps and weighed. Specific activity for glucose (d.p.m. pmol^−1^) was determined and data are expressed as picomoles per milligram of tissue per hour.

### Skeletal muscle glycogen content, citrate synthase activity and immunoblots

Glycogen contents and citrate synthase activities were determined in semitendinosus muscles collected at necropsy as described previously (Camacho *et al*. 2017). Glucose concentrations from the extracted glycogen were determined in triplicate, and results are expressed as milligrams of glucose per gram of tissue (wet weight). Citrate synthase activity was measured with the citrate synthase assay kit (Sigma‐Aldrich) from 20 µg of protein and are expressed as activity per microgram of protein.

Immunoblots were performed on protein lysates prepared from semitendinosus muscle (30–40 mg) with CelLytic MT Cell Lysis Reagent (Sigma‐Aldrich) and protease inhibitors (0.5 mM phenylmethylsulfonyl fluoride (PMSF), 2 µg ml^−1^ Aprotinin, 2.5 µg ml^−1^ Leupeptin) as described previously (Camacho *et al*. 2017). Primary antibodies used were raised in rabbit against glucose transporter 1 (GLUT1, 1:250, Millipore Cat. no. 07‐1401, lot no. 2630748, RRID:AB_1587074), glucose transporter 4 (GLUT4, 1 µg ml^−1^, Sigma‐Aldrich Cat. no. G4048, lot no. 016M4809V, RRID:AB_1840900), ADRβ2 (ADRB2; 1:250, Santa Cruz Biotechnology Cat. no. sc‐569, lot no. G3115, RRID:AB_630926), insulin receptor β‐subunit (INSR, 1:250, Santa Cruz Biotechnology Cat. no. sc‐711, lot no. H0916, RRID:AB_631835) and Tubulin‐β (TUBB; 1:1000, Thermo Fisher Scientific no. RB‐9249‐P0, lot no. 9249P1507B and 9249P1603L, RRID:AB_722289). Antibody complexes were detected with anti‐rabbit IgG horseradish peroxidase conjugated secondary antibody (1:15,000; Bio‐Rad Laboratories, Hercules, CA, USA) and chemiluminescence (West Pico Chemiluminescent Substrate; Thermo Fisher). Protein concentrations were quantified using photographed images and densitometry analyses (Scion Image Software, Frederick, MD, USA). Protein loading was normalized with Tubulin‐β concentrations. To accommodate the number of samples, two immunoblots were run simultaneously that contained overlapping samples for internal controls. Data are presented as percentage of the control mean.

### Statistical analysis

Lamb morphometric characteristics, citrate synthase activity, glycogen content, protein expression, arterial blood pressure, heart rate and dobutamine responses were analysed by ANOVA for group effects using the MIXED procedure of SAS 9.4 (SAS Institute, Cary, NC, USA). Differences were determined with a *post hoc* Fisher's least significant difference test. Growth rates and growth rates/birth weights were examined using repeated measures analysis of the MIXED procedure. Factors included in the repeated measure models were experimental group, time (day) and their interaction. Means were separated using the PDIFF option of the LSMEANS statement of SAS. The GSIS and arginine‐stimulated insulin secretion studies were analysed by ANOVA using the MIXED procedure. The model included experimental groups (control, IUGR and IUGR‐AD), draw time, and their interaction. Appropriate (minimize information criterion) covariance structures were selected using the best fit statistics. *In vivo* rates and fluxes were analysed by ANOVA using the MIXED procedure with lamb as the random effect. Main effects were experimental group (control, IUGR and IUGR‐AR lambs), study period defined by the insulin infusion rate (basal and hyperinsulinaemia) and their interaction. *Ex vivo* muscle glucose oxidation rates were analysed by ANOVA with the MIXED procedure for effects of experimental group, media condition (basal, insulin, insulin + catecholamines, or insulin + cytochalasin B), and their interaction. The main effect of sex was not tested due to insufficient power in the IUGR and IUGR‐AR groups. Means were separated using the Fisher's least significant difference test and were considered significant when α ≤ 0.05. In the absence of interactions (*P* > 0.05), significant main effects are reported; otherwise, interactive means are discussed. Lamb was considered the experimental unit for all outputs. Data are presented as the mean ± standard error of the mean.

## Results

### Morphometry at birth and postnatal growth

Morphometric measurements at birth, sex ratios and gestational lengths for lambs are presented in Table 1. At birth, IUGR and IUGR‐AR lambs were lighter, had shorter crown–rump lengths, and had reduced head circumference‐to‐body weight ratios than controls.

**Table 1:** Birth morphometry

Group (*n*)	Control (15)	IUGR (14)	IUGR‐AR (8)
Sex (M:F)	9:6	3:11	2:6
Gestational age (day)	151 ± 1^a^	149 ± 1^b^	148 ± 1^b^
Birth weight (kg)	4.3 ± 0.2^a^	2.9 ± 0.3^b^	2.5 ± 0.4^b^
Crown–rump length (cm)	49.7 ± 1.0^a^	43.3 ± 1.6^b^	41.6 ± 1.9^b^
Hindlimb length (cm)	40.9 ± 1.8	36.8 ± 2.2	38.6 ± 3.6
Head circumference (cm)	24.9 ± 0.7	22.4 ± 0.9	20.7 ± 0.8
Head circumference/weight	5.9 ± 0.2^a^	8.5 ± 0.7^b^	9.5 ± 1.1^b^

Animal numbers (*n*) within groups are presented in parentheses. Data are expressed as the mean ± SEM. Differences (*P* < 0.05) between groups are identified with different superscript letters.

**Figure 1:**
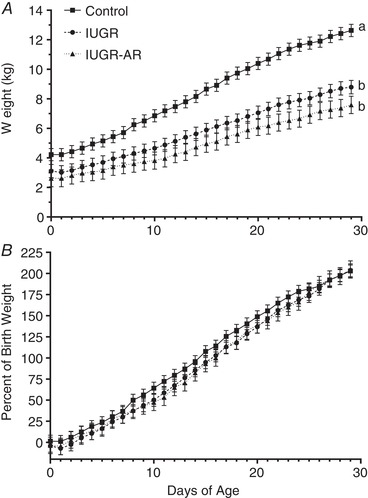
Lamb growth *A*, average daily weights are presented for control (*n* = 15), IUGR (*n* = 14) and IUGR‐AR (*n* = 8) lambs. *B*, daily weights as a percentage of birth weight are presented for lambs in each experimental group. Different letters on the right side of *A* indicate group differences.

Control lambs were heavier than IUGR and IUGR‐AR lambs throughout the 30‐day period (Fig. 1 *A*). Absolute growth rates were greater (*P* < 0.01) in control lambs (292 ± 14 g day^−1^) than in IUGR (209 ± 17 g day^−1^) or IUGR‐AR (175 ± 19 g day^−1^) lambs, but IUGR and IUGR‐AR growth rates were not different from each other. Growth as a percentage of birth weight was not different among groups (Fig. 1 *B*). Feed‐to‐weight gain efficiencies were not different among groups (0.77 ± 0.04, 0.78 ± 0.04 and 0.81 ± 0.05 g g^−1^ for control, IUGR and IUGR‐AR lambs, respectively). At 28 ± 1 days of age, IUGR (8.4 ± 0.6 kg) and IUGR‐AR (6.9 ± 1.0 kg) lambs weighed less (*P* < 0.05) than control lambs (12.5 ± 0.5 kg).

### Cardiovascular measurements

**Figure 2:**
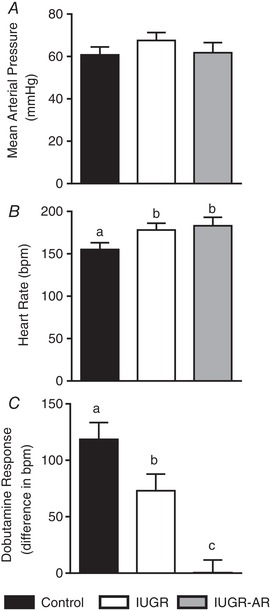
Mean arterial blood pressure and heart rates Mean arterial pressure (*A*) and heart rate (*B*) are presented for control (*n* = 12; 7 male (M)/5 female (F)), IUGR (*n* = 14; 3M/11F) and IUGR‐AR (*n* = 7; 1M/6F) lambs. Dobutamine response (*C*) was calculated as the difference in heart rate between stimulated and unstimulated periods for control (*n* = 4; 3M/1F), IUGR (*n* = 4; 2M/2F) and IUGR‐AR (*n* = 7; 1M/6F) lambs. Differences (*P* < 0.05) between experimental groups are indicated by different letters.

Cardiovascular measurements were conducted to confirm the functional presence of administered ADRβ pharmacological modulators. Mean arterial blood pressure was not different among experimental groups (Fig. 2 *A*). Resting heart rate was higher in IUGR and IUGR‐AR lambs compared to control lambs (Fig. 2 *B*). Administration of the ADRβ1 agonist dobutamine increased heart rates in control lambs by 118 ± 15 beats min^−1^ from basal, which was of greater magnitude (*P* < 0.05) than the 73 ± 15 beats min^−1^ increase in IUGR lambs. There was no response to dobutamine in IUGR‐AR lambs, which was expected due to the ongoing oral administration of the ADRβ1 inhibitor atenolol (Fig. 2 *C*).

### Glucose‐ and arginine‐stimulated insulin concentrations

**Figure 3:**
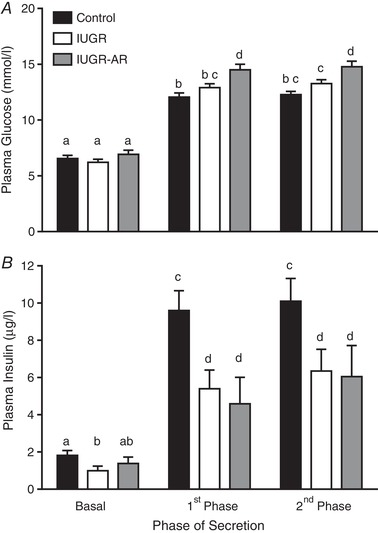
Glucose stimulated insulin concentrations Square‐wave hyperglycaemic clamps were performed in control (*n* = 12; 7M/5F), IUGR (*n* = 13; 3M/10F) and IUGR‐AR (*n* = 7; 1M/6F) lambs at 28 days of age. Mean (±SEM) plasma glucose (*A*) and insulin (*B*) concentrations are presented for basal (fasting), first‐phase (0–15 min) and second‐phase (20–60 min) periods of the GSIS study. The different letters above the bars represent significant difference between means (*P* < 0.05).

Fasting plasma glucose concentrations were not different among groups, and glucose and insulin concentrations increased (*P* < 0.05) during the hyperglycaemia clamp (Fig. 3). Fasting plasma insulin concentrations were lower in IUGR lambs compared to control lambs, but there was no difference between IUGR and IUGR‐AR groups. For first‐phase GSIS, glucose concentrations were greater in IUGR‐AR lambs compared to IUGR and control lambs, whereas, first‐phase insulin concentrations were lower in IUGR and IUGR‐AR lambs compared to control lambs. For second‐phase GSIS, glucose concentrations remained greater in IUGR‐AR lambs compared to IUGR and control lambs, but not different between IUGR and control lambs. Second‐phase insulin concentrations remained lower in IUGR lambs compared to control lambs, but insulin concentrations in IUGR‐AR lambs were intermediate to control and IUGR lambs. Glucose‐potentiated arginine‐stimulated insulin concentrations were similar among experimental groups (mean values for controls 31.8 ± 5.2 µg l^−1^, IUGR lambs 30.7 ± 4.5 µg l^−1^, IUGR‐AR lambs 27.4 ± 5.3 µg l^−1^).

Plasma cortisol concentrations were greater in IUGR‐AR lambs compared to IUGR and control lambs (Table 2). Plasma adrenaline and noradrenaline concentrations were not different among groups.

**Table 2:** Plasma cortisol and catecholamine concentrations

Group (*n*)	Control (12)	IUGR (13)	IUGR‐AR (7)
Cortisol (µg l^−1^)	22.8 ± 5.9^a^	24.4 ± 5.6^a^	46.2 ± 7.4^b^
Adrenaline (ng l^−1^)	109 ± 37	131 ± 17	102 ± 28
Noradrenaline (ng l^−1^)	573 ± 80	1076 ± 337	842 ± 304

Animal numbers (*n*) within groups are presented in parentheses. Data are expressed as the mean ± SEM. Differences (*P* < 0.05) between groups are identified with different superscript letters. Plasma samples were collected during the basal period of the GSIS study.

### Whole‐body insulin sensitivity for glucose utilization

**Figure 4:**
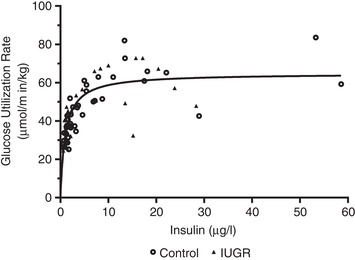
Whole‐body glucose utilization rate dose–response to insulin Body weight‐specific net glucose utilization rates were determined in control (*n* = 14; 8M/6F) and IUGR (*n* = 14; 3M/11F) lambs at 28 days of age. No differences were found between IUGR and control groups, and all lambs were combined to evaluate the dose–response relationship. Glucose utilization rates were measured at basal and three hyperinsulinaemic–euglycaemic periods by infusing insulin at 0.5, 2 and 4 mU min^−1^ kg^−1^. Each lamb was evaluated at two or three steady‐state periods. Insulin concentrations (µg l^−1^) are plotted on the *x*‐axis and net glucose utilization rates (µmol min^−1^ kg^−1^) on the *y*‐axis. A Michaelis–Menten equation (*R* ^2^ = 0.64) was used to predict maximum rate of glucose utilization (65 ± 2 µmol min^−1^ kg^−1^) and half‐maximum insulin concentration (1.0 ± 0.1 µg l^−1^) for glucose utilization rate in lambs.

The dose–response relationship between plasma insulin concentrations and whole‐body net glucose utilization rates was best fitted (*R* ^2^ = 0.64) by the Michaelis–Menten equation shown in Fig. 4, which estimated insulin responsiveness (maximum glucose utilization rate) at 65 ± 2 µmol min^−1^ kg^−1^ and insulin sensitivity (insulin concentration needed for half‐maximum glucose utilization rate) at 1.0 ± 0.1 µg l^−1^. Lambs from control and IUGR groups had similar blood glucose concentrations under all conditions (6.0 ± 0.2 mmol l^−1^). Plasma insulin concentrations and body weight‐specific net glucose utilization rates used to generate the dose–response curves were not different between control (*V*_max_ 68 ± 3 µmol min^−1^ kg^−1^, *K*_m_ 1.3 ± 0.2 µg l^−1^) and IUGR lambs (*V*_max_ 63 ± 3, *K*_m_ 0.9 ± 0.2) at any infusion rate, and these two groups were combined to analyse this relationship. Plasma insulin concentrations also did not differ between basal and 0.5 mU min^−1^ kg^−1^ infusion rates (1.5 ± 0.2 and 3.1 ± 1.2 µg l^−1^, respectively) among groups. However, plasma insulin concentrations increased (*P* < 0.01) sequentially at 2 and 4 mU min^−1^ kg^−1^ infusion rates (8.4 ± 1.3 and 20.5 ± 3.2 µg l^−1^, respectively). Whole‐body glucose utilization rates increased (*P* ≤ 0.05) sequentially from the basal period (35 ± 1 µmol min^−1^ kg^−1^) to each subsequent insulin infusion rate at 0.5 mU min^−1^ kg^−1^ (39 ± 2 µmol min^−1^ kg^−1^), 2 mU min^−1^ kg^−1^ (52 ± 2 µmol min^−1^ kg^−1^) and 4 mU min^−1^ kg^−1^ (65 ± 2 µmol min^−1^ kg^−1^).

**Figure 5:**
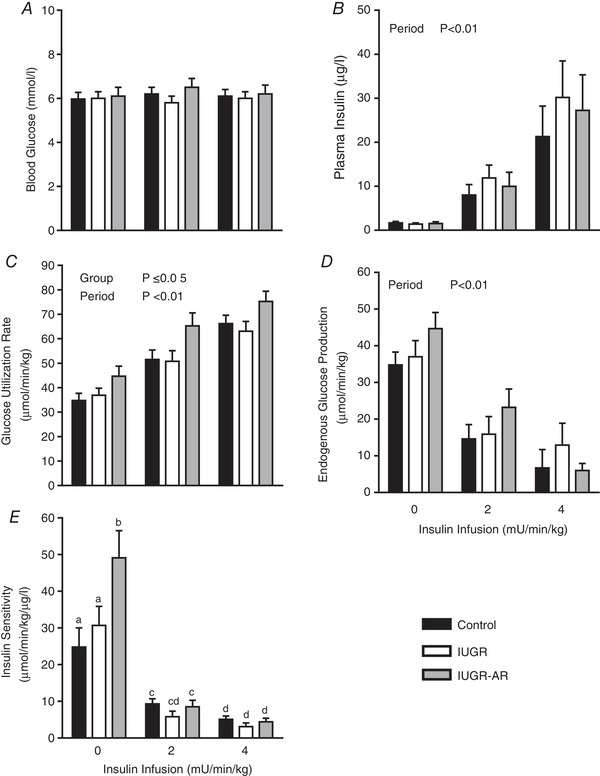
Whole‐body glucose utilization rates Hyperinsulinaemic–euglycaemic clamps were performed in control (*n* = 14; 8M/6F), IUGR (*n* = 14; 3M/11F) and IUGR‐AR (*n* = 7; 1M/6F) lambs at 28 days of age. Whole‐body net glucose utilization rates were measured at basal and two hyperglycaemic periods created with sequential increases in the insulin infusion rate (0, 2 and 4 mU min^−1^ kg^−1^), which are indicated on the *x*‐axis. Experimental group means ± SEM are presented for blood glucose concentrations (*A*), plasma insulin concentrations (*B*), body weight‐specific glucose utilization rates (*C*), endogenous glucose production rates (*D*) and insulin sensitivity (*E*). Main effects in the two‐way ANOVA included experimental group, study period for the insulin infusion rate, and their interaction (group by period). *P* values are presented for these effects if they were significant (*P* ≤ 0.05). For an interaction, differences (*P* ≤ 0.05) are identified by different letters.

Based on the dose–response curve, insulin infusion rates of 2 and 4 mU min^−1^ kg^−1^ were evaluated to determine insulin sensitivity for whole‐body net glucose utilization rates. No interactions were found between groups or insulin infusion rate for glucose concentrations, insulin concentrations, glucose utilization rates, or endogenous glucose production rates, which indicates that all lambs had similar responses to the increased insulin infusion rates. Blood glucose concentrations were not different among all groups or between the different insulin infusion rates (Fig. 5 *A*). Plasma insulin concentrations were not different among groups within any infusion period, but average insulin concentrations across all groups increased (*P* < 0.01) from basal (1.5 ± 0.2 µg l^−1^), as expected, when insulin was infused at 2 mU min^−1^ kg^−1^ (10.0 ± 1.7 µg l^−1^) and 4 mU min^−1^ kg^−1^ (26.2 ± 4.5 µg l^−1^; Fig. 5 *B*). Regardless of the insulin infusion rate during the study, whole‐body net glucose utilization rates were greater in IUGR‐AR lambs (62 ± 4 µmol min^−1^ kg^−1^) than in IUGR (50 ± 3 µmol min^−1^ kg^−1^) and control lambs (51 ± 3 µmol min^−1^ kg^−1^; Fig. 5 *C*). Endogenous glucose production rates decreased from basal periods (39 ± 2 µmol min^−1^ kg^−1^) when insulin was infused at 2 mU min^−1^ kg^−1^ (18 ± 3 µmol min^−1^ kg^−1^) or 4 mU min^−1^ kg^−1^ (10 ± 3 µmol min^−1^ kg^−1^), but were not different among groups for either insulin infusion rate (Fig. 5 *D*). There was a group by insulin infusion rate interaction (*P* < 0.05) for insulin sensitivity (Fig. 5 *E*); under basal conditions, insulin sensitivity was greater in IUGR‐AR lambs than in control or IUGR lambs. During either hyperinsulinaemic period, insulin sensitivity was lower than at basal conditions but was not different among all groups.

### Hindlimb metabolic fluxes

Arterial blood oxygen content, plasma glucose and lactate concentrations, and hindlimb oxygen uptake rates, lactate output rates and nutrient oxygen quotients measured during the hyperinsulinaemic–euglycaemic clamps are presented in Table 3. No interaction between experimental groups and insulin infusion rates was observed for any of these variables. Plasma glucose concentrations, blood oxygen content and hindlimb net oxygen uptake rates were not different among groups or at the two insulin infusion rates. Plasma lactate concentrations were similar between IUGR lambs (0.73 ± 0.08 mmol l^−1^) and IUGR‐AR lambs (0.92 ± 0.08 mmol l^−1^) but were greater (*P* < 0.05) than in control lambs (0.50 ± 0.07 mmol l^−1^). Hindlimb lactate output rates and lactate oxygen quotients were not different among groups or at the two insulin infusion rates.

**Table 3:** Hindlimb metabolic flux measurements for arterial oxygen, lactate and glucose concentrations, oxygen and lactate uptakes, and metabolic quotients

	Insulin infusion rate											
	0 mU min^−1^ kg^−1^			2 mU min^−1^ kg^−1^			4 mU min^−1^ kg^−1^			*P* values		
Experimental group	Control	IUGR	IUGR‐AR	Control	IUGR	IUGR‐AR	Control	IUGR	IUGR‐AR	G	P	I
Hindlimb blood flow (ml min^−1^)	55 ± 4	46 ± 5	47 ± 5	53 ± 6	38 ± 2	44 ± 7	52 ± 6	44 ± 5	46 ± 5	0.31	0.10	0.26
Hindlimb blood flow (ml min^−1^ kg^−1^)	45 ± 4	64 ± 7	64 ± 6	43 ± 5	54 ± 9	62 ± 7	42 ± 5	62 ± 10	61 ± 7	0.08	0.11	0.28
Blood oxygen (mmol l^−1^)	7.0 ± 0.2	6.8 ± 0.3	6.2 ± 0.5	6.9 ± 0.3	6.8 ± 0.4	6.6 ± 0.3	7.0 ± 0.3	6.8 ± 0.3	6.2 ± 0.5	0.25	0.95	0.86
Oxygen uptake (µmol min^−1^ kg^−1^)	103 ± 14	131 ± 15	124 ± 5	109 ± 12	138 ± 24	149 ± 15	106 ± 12	153 ± 36	126 ± 13	0.14	0.28	0.42
Plasma lactate (mmol l^−1^)	0.52 ± 0.04	0.60 ± 0.08	1.01 ± 0.16	0.45 ± 0.03	0.71 ± 0.10	0.74 ± 0.09	0.54 ± 0.04	0.87 ± 0.19	0.98 ± 0.20	<0.01	0.19	0.45
Lactate uptake (µmol min^−1^ kg^−1^)	−6.9 ± 0.9	−8.5 ± 1.4	−11.3 ± 2.1	−6.8 ± 1.0	−12.7 ± 4.2	−11.6 ± 2.3	−7.3 ± 1.5	−11.7 ± 2.4	−12.0 ± 1.1	0.10	0.14	0.44
Lactate O_2_ quotient	−0.22 ± 0.04	−0.21 ± 0.04	−0.27 ± 0.04	−0.20 ± 0.04	−0.28 ± 0.09	−0.23 ± 0.04	−0.22 ± 0.04	−0.22 ± 0.03	−0.29 ± 0.01	0.63	0.92	0.26
Plasma glucose (mmol l^−1^)	6.5 ± 0.1	7.1 ± 0.9	6.8 ± 0.5	6.6 ± 0.1	6.7 ± 0.7	7.1 ± 0.3	6.6 ± 0.1	6.8 ± 0.6	6.7 ± 0.6	0.79	0.39	0.17
Glucose extraction eff. (%)	4.6 ± 0.5	5.2 ± 0.9	3.8 ± 0.6	6.0 ± 0.6	8.9 ± 0.8	6.7 ± 1.3	11.2 ± 1.0	10.7 ± 1.9	9.2 ± 1.4	0.29	<0.01	0.47
Glucose O_2_ quotient	0.78 ± 0.14	0.94 ± 0.18	0.72 ± 0.19	1.04 ± 0.05	1.29 ± 0.16	1.15 ± 0.36	1.64 ± 0.08	1.54 ± 0.28	1.56 ± 0.27	0.79	<0.01	0.79
G + L O_2_ quotient	0.55 ± 0.11	0.73 ± 0.15	0.45 ± 0.16	0.83 ± 0.02	1.01 ± 0.17	0.92 ± 0.33	1.43 ± 0.05	1.32 ± 0.28	1.27 ± 0.26	0.71	<0.01	0.81

Data are presented as the mean ± SEM for 7 control lambs, 5 IUGR lambs and 5 IUGR‐AR lambs. *P* values for the ANOVA analysis on group (G), insulin infusion rate (study period, P) and the interaction (group × infusion rate; I) are present. Glucose extraction eff., glucose extraction efficiency; G + L, glucose + lactate.

**Figure 6:**
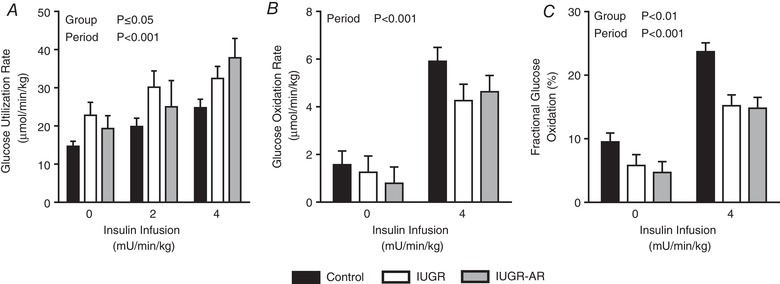
Hindlimb glucose fluxes Hindlimb weight‐specific net glucose utilization rates (*A*), glucose oxidation rates (*B*) and fractional glucose oxidation (*C*) were measured in control (*n* = 7; 3M/4F), IUGR (*n* = 5; 1M/4F) and IUGR‐AR (*n* = 5; 1M/4F) lambs. Rates of glucose utilization across the hindlimb were determined at three steady‐state periods: basal (0 mU min^−1^ kg^−1^) and two hyperinsulinaemic periods produced with insulin infusion rates at 2 and 4 mU min^−1^ kg^−1^ (*x*‐axis). Glucose oxidation and fractional glucose oxidation rates were evaluated at basal and 4 mU min^−1^ kg^−1^ infusion periods. Main effects in the two‐way ANOVA included experimental group, study period for the insulin infusion rate and their interaction (group by period). *P* values are presented on the graph if the effects were significant (*P* ≤ 0.05).

Hindlimb glucose utilization rates were not different between IUGR and IUGR‐AR lambs (28.4 ± 2.9 and 28.6 ± 3.0 µmol min^−1^ kg^−1^, respectively), but hindlimb glucose utilization rates in both experimental groups were greater (*P* < 0.05) than control lambs (19.8 ± 2.5 µmol min^−1^ kg^−1^), regardless of the insulin infusion rate (Fig. 6 *A*). Across all groups, hindlimb glucose utilization rates increased from basal (18.9 ± 1.9 µmol min^−1^ kg^−1^) to hyperinsulinaemic periods when insulin was infused at 2 mU min^−1^ kg^−1^ (25.9 ± 1.9 µmol min^−1^ kg^−1^) and 4 mU min^−1^ kg^−1^ (32.0 ± 3.0 µmol min^−1^ kg^−1^). This coincided with greater glucose extraction efficiencies (4.5 ± 0.6%, 7.1 ± 0.6% and 10.4 ± 0.6% at basal, and 2 and 4 mU min^−1^ kg^−1^, respectively) and glucose oxygen quotients (0.81 ± 0.11, 1.13 ± 0.11 and 1.59 ± 0.11 at basal, and 2 and 4 mU min^−1^ kg^−1^, respectively; Table 3). Glucose extraction efficiencies, glucose oxygen quotients, and glucose + lactate oxygen quotients were not different among groups. However, glucose + lactate oxygen quotient increased sequentially with higher insulin infusion rates (0.58 ± 0.10, 0.89 ± 0.11, 1.34 ± 0.11 at 0, 2 and 4 mU min^−1^ kg^−1^). Hindlimb glucose oxidation rates increased from basal (1.2 ± 0.4 µmol min^−1^ kg^−1^) to subsequent periods when insulin was infused at 4 mU min^−1^ kg^−1^ (4.9 ± 0.4 µmol min^−1^ kg^−1^), but were not different among groups during basal or hyperinsulinaemic periods (Fig. 6 *B*). However, hindlimb fractional glucose oxidation rates were lower in IUGR lambs (10.5 ± 1.4%) and IUGR‐AR lambs (9.7 ± 1.4%) than in control lambs (16.6 ± 1.2%) regardless of insulin infusion rate (Fig. 6 *C*). In all groups, fractional glucose oxidation rates increased from basal (6.7 ± 1.0%) to subsequent higher insulin infusion rates (17.9 ± 1.0%).

### Post‐mortem organ weights

At necropsy body weight and brain, lung, liver, kidney, heart, left ventricle, right ventricle and hindlimb weights were less in IUGR and IUGR‐AR lambs compared to controls (Table 4). Brain and liver weights relative to body weights were greater in IUGR and IUGR‐AR lambs compared to control lambs. Lung, kidney and right ventricle weights relative to body weight were not different among groups. Heart weight relative to body weight was greater in IUGR‐AR lambs than in IUGR and control lambs, and relative left ventricle weight was greater in IUGR‐AR lambs than controls.

**Table 4:** Post‐mortem organ weights

Group (*n*)	Control (15)	IUGR (14)	IUGR‐AR (7)
Body weight (kg)	13.1 ± 0.6^a^	8.9 ± 0.6^b^	7.3 ± 0.8^b^
Brain (g)	73 ± 2^a^	64 ± 2^b^	62 ± 3^b^
Lung (g)	229 ± 11^a^	163 ± 12^b^	127 ± 16 ^b^
Liver (g)	342 ± 15^a^	251 ± 15^b^	225 ± 21^b^
Average kidney (g)	40.3 ± 1.8^a^	30.3 ± 1.8^b^	25.6 ± 2.6^b^
Heart (g)	82 ± 4^a^	56 ± 4^b^	56 ± 6^b^
Right ventricle (g)	17.8 ± 1.0^a^	11.7 ± 1.0^b^	10.2 ± 1.4^b^
Left ventricle (g)	30.9 ± 1.7^a^	22.3 ± 1.9^b^	20.6 ± 2.5^b^
Hindlimb (kg)	1.20 ± 0.06^a^	0.80 ± 0.05^b^	0.68 ± 0.08^b^
Relative brain weight (g kg^−1^)^†^	5.6 ± 0.5^a^	7.8 ± 0.5^b^	9.6 ± 0.8^b^
Relative lung weight (g kg^−1^)^†^	17.5 ± 0.7	18.8 ± 0.7	18.0 ± 1.0
Relative liver weight (g kg^−1^)^†^	26.1 ± 0.9^a^	29.0 ± 0.9^b^	31.7 ± 1.3^b^
Relative kidney weight (g kg^−1^)^†^	3.1 ± 0.2	3.5 ± 0.2	3.8 ± 0.3
Relative heart weight (g kg^−1^)^†^	6.2 ± 0.2^a^	6.3 ± 0.2^a^	7.8 ± 0.3^b^
Relative left ventricle weight (g kg^−1^)^†^	2.4 ± 0.1^a^	2.6 ± 0.1^ab^	2.8 ± 0.2^b^
Relative right ventricle weight (g kg^−1^)^†^	1.4 ± 0.1	1.3 ± 0.1	1.4 ± 0.1

Data are expressed as the mean ± SEM. Differences (*P* < 0.05) between groups are identified with different superscript letters. ^†^Relative to body weight.

### *In vitro* pancreatic islet insulin secretion

**Figure 7:**
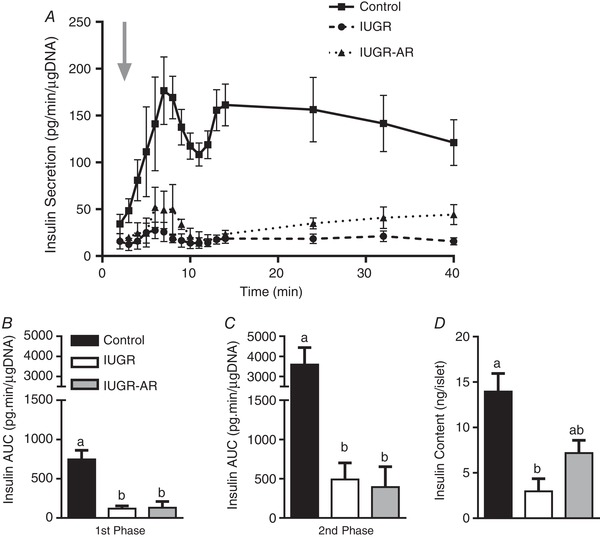
Isolated islet insulin secretion responsiveness to glucose Dynamic measurements of glucose‐stimulated insulin secretion were analysed in islets isolated from control (*n* = 4; 2M/2F), IUGR (*n* = 3; 1M/2F) and IUGR‐AR (*n* = 3; 0M/3F) lambs. *A*, insulin secretion rates (pg min^−1^ (µg DNA)^−1^) are shown at the time collected. Stimulatory glucose concentrations (11.1 mmol l^−1^) were initiated at 3 min, which is indicated by the grey arrow. *B*, area under the curve (AUC) for 3–11 min was calculated for each experimental group to measure first‐phase insulin secretion. *C*, AUCs for second‐phase insulin secretion (12–40 min) are presented. *D*, islet insulin contents were measured. Differences (*P* < 0.05) between experimental groups are indicated by different letters.

Pancreatic islet viability assessed by OCR/DNA was not different between control (387 ± 23 nmol O_2_ min^−1^ (mg DNA)^−1^) and IUGR lambs (396 ± 43 nmol O_2_ min^−1^ (mg DNA)^−1^). Islets from IUGR and IUGR‐AR lambs had reduced first‐phase and second‐phase GSIS compared to islets from control lambs (Fig. 7). IUGR and IUGR‐AR GSIS response was similar. Islets from IUGR lambs also had less insulin content compared to control islets, but IUGR‐AR islets were not different among groups.

### *Ex vivo* skeletal glucose oxidation rates

**Figure 8:**
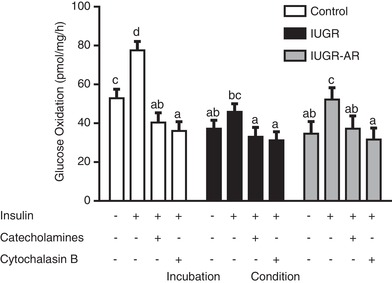
*Ex vivo* glucose oxidation rates in skeletal muscle Glucose oxidation rates were measured in semitendinosus muscle fibres from control (*n* = 12; 8M/4F), IUGR (*n* = 13; 3M/10F) and IUGR‐AR (*n* = 6; 1M/5F) lambs. Rates of glucose oxidation were determined in four media conditions containing no hormones (basal), 10 µU ml^−1^ insulin, insulin (10 µU ml^−1^) + catecholamines (12.5 µmol l^−1^ adrenaline and 12.5 µmol l^−1^ noradrenaline) and insulin (10 µU ml^−1^) + 20 mmol l^−1^ cytochalasin B. Differences (*P* ≤ 0.05) are identified by different letters above the bars (means ± SEM).

There was an interaction (*P* < 0.05) between experimental groups and incubation media for *ex vivo* glucose oxidation rates in primary myofibres. Glucose oxidation rates of muscle fibres from control lambs were 25 ± 5 pmol mg^−1^ h^−1^ greater in media with insulin compared to basal media without insulin (Fig. 8). Inclusion of cytochalasin B (glucose transport inhibitor) or catecholamines to insulin‐supplemented media decreased insulin‐stimulated glucose oxidation rates in muscle fibres from controls to rates that were below those observed in basal media. Glucose oxidation rates in basal media were not different between IUGR and IUGR‐AR muscle fibres but were lower in both of these groups compared to control muscle. Insulin‐stimulated glucose oxidation rates were not different between IUGR and IUGR‐AR muscle fibres but were 33 ± 6 and 27 ± 8 pmol mg^−1^ h^−1^ lower, respectively, compared to control muscle fibres. Moreover, insulin‐supplemented media had no effect on glucose oxidation rates in IUGR muscle fibres when compared to rates in basal media, but insulin increased glucose oxidation rates 18 ± 7 pmol mg^−1^ h^−1^ above basal rates in IUGR‐AR muscle fibres. Inclusion of cytochalasin B or catecholamines in insulin‐supplemented media reduced glucose oxidation rates in IUGR and IUGR‐AR muscle fibres to rates that were not different from those observed in basal media.

### Skeletal muscle glycogen content and citrate synthase activity

Glycogen content in the semitendinosus muscle was not different among control (13.7 ± 1.2 mg g^−1^), IUGR (15.3 ± 1.1 mg g^−1^) and IUGR‐AR (11.9 ± 1.2 mg g^−1^) groups. Citrate synthase activities were not different between semitendinosus muscles from control (343 ± 13 µmol min^−1^ (mg protein)^−1^) and IUGR lambs (348 ± 12 µmol min^−1^ (mg protein)^−1^) but were lower (*P* < 0.05) in IUGR‐AR lambs (287 ± 15 µmol min^−1^ (mg protein)^−1^) compared to control or IUGR lambs.

### Glucose transporters, insulin receptor and ADRβ2 in muscle

**Figure 9:**
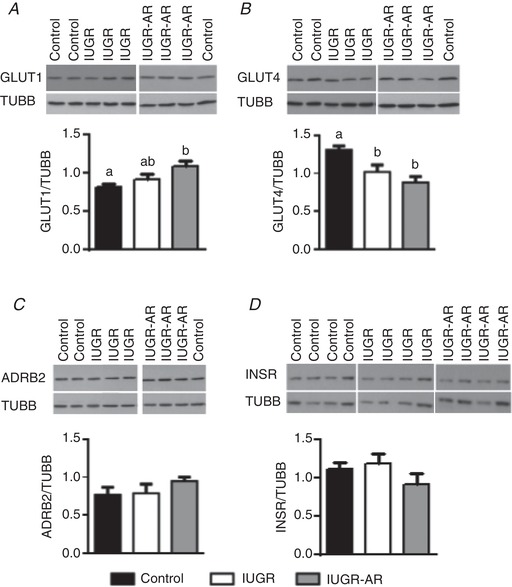
Glucose transporters, insulin receptor and adrenergic receptor β2 expression in skeletal muscle Protein expression levels of GLUT1 (*A*), GLUT4 (*B*), ADRβ2 (ADRB2; *C*) and insulin receptor β (INSR; *D*) were measured in semitendinosus muscle from control (*n* = 8; 4M/4F), IUGR (*n* = 8; 3M/5F) and IUGR‐AR (*n* = 7; 1M/6F) lambs. Representative images of western blots are shown for the glucose transporters and receptors and β‐Tubulin (TUBB), which was used for normalization. Means ± SEM are presented in the bar graphs and differences (*P* < 0.05) are indicated with different letters.

GLUT1 concentrations in the semitendinosus muscle were not different between control and IUGR lambs or between IUGR and IUGR‐AR lambs but were greater (*P* < 0.05) in IUGR‐AR lambs compared to control lambs (Fig. 9 *A*). GLUT4 concentrations were lower (*P* < 0.05) in the semitendinosus muscle from IUGR and IUGR‐AR lambs than in control lambs (Fig. 9 *B*). Protein concentrations for ADRβ2 and INSR were not different among groups (Fig. 9 *C* and *D*).

## Discussion

Previous work has shown that IUGR fetal sheep produced by placental insufficiency develop disparities in insulin secretion, skeletal muscle growth and glucose metabolism in late gestation (Limesand *et al*. 2006, 2007; Brown *et al*. 2015; Yates *et al*. 2016). In this study, we show that the disruption in pancreatic islet insulin secretion and skeletal muscle glucose metabolism persisted in 1‐month‐old lambs that were born following placental insufficiency‐induced IUGR, despite normal whole‐body glucose utilization rates and insulin sensitivity. Specifically, we found that GSIS, which is enhanced in IUGR lambs at earlier ages (Camacho *et al*. 2017), was reduced at 1 month of age despite normal plasma catecholamine concentrations at this age. Reductions in insulin secretion responsiveness in IUGR lambs were associated with less islet insulin content, which indicates a diminished capacity for their β‐cells to synthesize and store insulin, similar to the IUGR fetus (Limesand *et al*. 2006). Additionally, the IUGR hindlimb glucose utilization rates were higher than normal but the fractional glucose oxidation rates were lower. Studies in primary muscle fibres from IUGR lambs further showed that the muscle‐specific glucose oxidative capacity was impaired. Postnatal manipulation of β adrenergic receptor activity via daily oral treatment with ADRβ2 agonist and ADRβ1/β3 antagonists improved whole‐body glucose utilization rates and basal insulin sensitivity in IUGR lambs. However, the treatment had no effect on their hindlimb glucose metabolism or skeletal muscle glucose oxidation rates. This coincided with equivalent skeletal muscle expression of ADRβ2 and insulin receptor β among all groups. Unexpectedly, IUGR lambs also had faster heart rates that were independent of ADRβ1 activation because postnatal ADRβ1 antagonists did not slow heart rates in IUGR‐AR lambs. The findings from this study show that changes in β adrenergic activity underlie some but not all postnatal outcomes of IUGR produced by placental insufficiency and that multiple mechanisms contribute to the programming of metabolic dysfunction in the IUGR lambs.

### Neonatal growth

At birth, IUGR lambs weighed less than controls and had asymmetric fetal growth restriction, which is consistent with fetal growth patterns and birth metrics for pathological IUGR human infants (Riyami *et al*. 2011). IUGR lambs had greater head circumference‐to‐birth weight ratios that indicate fetal brain sparing, which was confirmed at necropsy. This asymmetry was comparable to the ovine endometrial carunclectomy model of placental restriction (De Blasio *et al*. 2006, 2007; Owens *et al*. 2007b *b*; Morrison, 2008; Liu *et al*. 2015). We attribute the asymmetric growth in our IUGR lambs to the progressive rise in circulating fetal catecholamines brought on by placental insufficiency‐induced hypoxaemia and hypoglycaemia (Macko *et al*. 2013, 2016; Davis *et al*. 2015). Although not measured in the present study, it is reasonable to assume that our IUGR lambs were exposed to chronically elevated concentrations of catecholamines *in utero*, as shown previously (Limesand *et al*. 2006, 2013).

Infants with fetal growth restriction typically exhibit accelerated early‐life growth velocity, which independently predicts their risk for developing metabolic disease (Barker *et al*. 2002; Dulloo, 2006; Claris *et al*. 2010). Driven by greater fat deposition, early catch‐up growth is associated with greater central obesity, insulin resistance and cardiovascular disease in humans (Jaquet *et al*. 2000; Gluckman *et al*. 2008; Ibanez *et al*. 2008). In other sheep models of IUGR, longitudinal studies show that IUGR lambs reach normal body weights by 1 month of age (De Blasio *et al*. 2006; Liu *et al*. 2015; Spiroski *et al*. 2018). However, greater central adiposity at 6 weeks of age indicates that a larger percentage of their early weight gain is due to fat deposition, even though body composition normalized in adulthood (Liu *et al*. 2015). Similarly, the over‐nourished adolescent model of IUGR exhibited greater daily weight gain and adiposity near weaning, but their body composition normalized later (Wallace *et al*. 2018; Wallace, 2019). Unlike the other ovine models, our IUGR lambs had similar fractional growth rates through the first 30 days. The reason for the absence of catch‐up growth is not clear, but it is unlikely that it was due to insufficient postnatal nutrition. After colostrum, all lambs were fed similar amounts of commercial milk replacer to minimize nutrient variability from heat stressed ewes (Abdalla *et al*. 1993). The average daily gain for our control lambs was comparable to those reported in lambs reared by the ewe (Louey *et al*. 2000; De Blasio *et al*. 2006; Liu *et al*. 2015; Wallace *et al*. 2018). The lack of early‐stage catch‐up growth is in fact a major benefit of this model and study because early catch‐up growth may contribute to metabolic deficits in some cases. Here we demonstrate that metabolic deficits can occur in the absence of confounding early catch‐up growth. Therefore, despite the absence of confounding catch‐up growth in our IUGR lambs, defects in β‐cell function and skeletal muscle glucose metabolism were apparent, which reflect developmental programming due to the *in utero* environment.

The lack of improved growth performance in IUGR lambs treated with clenbuterol was unexpected based on previous findings in older lambs (Beermann *et al*. 1987; Bohorov *et al*. 1987). Beermann (2002) postulated that this unresponsiveness to an ADRβ2 agonist in young lambs is caused by either low skeletal muscle sensitivity to ADRβ2 or the growth rate is already near the maximum for skeletal muscle at this age. Our findings are inconsistent with ADRβ2 insensitivity because we found greater whole‐body glucose utilization rates and insulin sensitivity in IUGR‐AR lambs. However, if the latter is true and muscle growth is near maximum, repartitioning of nutrients with ADRβ2 would not be effective. Furthermore, younger, less physiologically mature animals have lower rates of lipid accretion than mature animals, which also lowers ADRβ2 agonist effectiveness (Williams *et al*. 1987; Maltin *et al*. 1990). Alternatively, the growth rates exhibited in our IUGR lambs may be indicative of a lower capacity for muscle accretion, as previously observed (Yates *et al*. 2014, 2016; Soto *et al*. 2017).

### Insulin secretion

At 1 month of age, IUGR lambs exhibited substantially impaired first‐ and second‐phase GSIS. Concurrent deficits in both phases of secretion are indicative of glucose intolerance, which frequently progresses with age to a diabetic state that is characterized by complete loss of the first‐phase secretion and further decline of the second‐phase secretion (Gerich, 2002; Seino *et al*. 2011). Lower GSIS by 1 month of age is a stark difference to the compensatory enhancement of GSIS that we previously observed in IUGR lambs at 1 week of age. In fetal sheep, placental insufficiency is associated with lower insulin secretion as early as 0.7 of gestation, which is prior to the onset of fetal growth restriction (Limesand *et al*. 2013; Macko *et al*. 2013). Blunted insulin secretion continues throughout the remainder of gestation and is inhibited in part by high concentrations of plasma catecholamines (Owens *et al*. 2007a; Leos *et al*. 2010; Macko *et al*. 2016). In fact, similar inhibition occurred when hypercatecholaminaemia was induced in normally grown fetuses with an exogenous noradrenaline infusion (Chen *et al*. 2014), acute maternofetal hypoxia (Jackson *et al*. 2000; Yates *et al*. 2012b), anaemic hypoxaemia (Benjamin *et al*. 2017), or insulin imbalance (Andrews *et al*. 2015). Interestingly, an acute adrenergic blockade in the IUGR fetus revealed the development of this hyper‐insulin secretion response (Leos *et al*. 2010; Macko *et al*. 2013). This compensatory enhancement of insulin secretion develops in opposition to chronic adrenergic stimulation, because we also observed hypersecretion in fetal sheep immediately after stopping a week‐long infusion of noradrenaline that was still present 5 days after the infusion was terminated (Chen *et al*. 2014, 2017). Primary islets from noradrenaline‐infused fetuses exhibited greater GSIS *in vitro*. These islets had normal insulin content, calcium signalling and morphology but showed evidence of adrenergic desensitization, which now appears to be the mechanism for compensatory insulin stimulus–secretion coupling (Chen *et al*. 2017; Kelly *et al*. 2018). Additionally, GSIS remained augmented in placental insufficiency‐induced, overnourished adolescent‐induced and twinning‐induced IUGR lambs for more than a week after birth (Gatford *et al*. 2013; Camacho *et al*. 2017; Wallace *et al*. 2018), which helps to explain the dangerous condition of transitional hyperinsulinaemic hypoglycaemia that frequently afflicts newborns with IUGR (Stanley *et al*. 2015; Rozance & Hay, 2016).

Our study shows that the compensatory enhancement of insulin secretion is a transient condition that subsides after the first few weeks of life to reveal programmed deficits in β‐cell function. Perfusion studies recapitulate the poor insulin secretion response of IUGR islets to glucose, demonstrating that the impairment is intrinsic to the β‐cells. In addition to lower insulin secretion responsiveness, the capacity of the IUGR islets to synthesize and store insulin was reduced, a deficiency that was shown previously in fetal sheep with placental insufficiency‐induced IUGR (Limesand *et al*. 2006). Although adrenergic dysregulation was shown to affect fetal islets with IUGR, daily administration of ADRβ modifiers did not improve islet response to glucose in IUGR lambs. However, there was a modest recovery in islet insulin content, which may reflect lower insulin demands due to the improved insulin sensitivity in IUGR‐AR lambs (Leos *et al*. 2010; Kelly *et al*. 2018). These findings show that pancreatic islet dysfunction persists after birth due to limited insulin production and storage in IUGR islets, even though there is hyper‐insulin secretion at earlier ages.

### Insulin action on tissues

Whole‐body glucose utilization rates were similar between control and IUGR lambs. Thus, data from both groups were pooled to construct dose–response curves for insulin‐stimulated glucose utilization, which were used to identify half‐maximal insulin sensitivity (ED_50_) and maximum insulin responsiveness as previously described (Kahn, 1978). Half‐maximal insulin sensitivity at 1 month of age was comparable to that observed in non‐pregnant adult ewes (Bergman *et al*. 1989; Petterson *et al*. 1993). Although neonatal ED_50_ was similar to adult ewes, maximum insulin responsiveness was about fourfold greater in lambs, which is consistent with the previously observed progressive decline in insulin action as sheep advance in age (Gatford *et al*. 2004).

We previously found that whole‐body glucose utilization rates were greater in IUGR lambs at 2 weeks of age (Camacho *et al*. 2017). In this study, similar whole‐body glucose utilization rates between control and IUGR lambs indicate that a correction in whole‐body insulin action for glucose occurs by 1 month of age. Furthermore, hepatic glucose production rates did not differ between control and IUGR lambs at 2 weeks (Camacho *et al*. 2017) or 1 month of age, even though plasma lactate concentrations were higher in IUGR lambs at both ages. The correction in whole‐body insulin action was not explained by hindlimb glucose fluxes or skeletal muscle glucose transporter profiles. Hindlimb glucose utilization rates were greater in IUGR lambs under basal and hyperinsulinaemic conditions, despite less GLUT4 and similar GLUT1 concentrations in skeletal muscle. Interestingly, there was no difference in GLUT1 or GLUT4 concentrations between control and IUGR skeletal muscle near term (Limesand *et al*. 2007) or at 2 weeks of age (Camacho *et al*. 2017). Therefore, muscle adaptations responsible for increased insulin sensitivity appear to be independent of the expression of these glucose transporters, but could involve the translocation of GLUT4 to the plasma membrane. Furthermore, these data indicate that compensatory changes in glucose uptake by other tissues might help to normalize glucose tolerance in IUGR lambs. These findings show continuing changes in glucose metabolism of IUGR lambs over the first month of life, although it is unclear whether changes in insulin sensitivity will ultimately progress into glucose intolerance as in other models (Ford *et al*. 2007; Owens *et al*. 2007b).

Whole‐body insulin sensitivity and glucose utilization rates were greater in IUGR lambs treated with β adrenergic modifiers. Across all insulin concentrations, whole‐body glucose utilization rates were ∼23% higher in IUGR‐AR lambs compared to both control and IUGR lambs. This coincided with greater hindlimb‐specific glucose utilization rates and skeletal muscle GLUT1 concentrations compared to control lambs. However, neither glucose utilization rates nor GLUT1 concentrations differed from untreated IUGR lambs, which indicates contributions from other mechanisms. In adult humans and rats, chronic administration of ADRβ2 agonists increased insulin sensitivity for non‐oxidative glucose utilization and lactate production but not glycogen synthesis (Scheidegger *et al*. 1984; Budohoski *et al*. 1987; Jensen *et al*. 2005). A subsequent study of post‐receptor interactions between insulin and β adrenergic signalling pathways via acute *ex vivo* treatments found that protein kinase A and protein kinase B (AKT) regulate distinct pools of glycogen synthase kinase‐3α/β that are separated by locale within the cell or by the niche of co‐activators (Jensen *et al*. 2007). We previously found that co‐incubation of rat soleus with insulin and ADRβ2 agonist for 1 h had an additive effect on AKT phosphorylation, although ADRβ2 agonist in the absence of insulin had no effect on AKT phosphorylation and in fact lowered glucose uptake (Cadaret *et al*. 2017). Additive post‐receptor effects may help to explain differences in acute *ex vivo* insulin‐stimulated glucose oxidation, but reduced citrate synthase activity associated with β adrenergic modifiers in our IUGR lambs indicate potentially reduced mitochondrial density and other detrimental metabolic changes.

Fractional glucose oxidation rates are lower in IUGR fetal sheep near term (Limesand *et al*. 2007; Brown *et al*. 2015), which we have postulated is due to skeletal muscle‐specific programming aimed at nutrient sparing (Yates *et al*. 2012a, 2018). In this study, we found that impaired glucose oxidative capacity was indeed muscle‐specific and persisted in IUGR lambs at 1 month of age, but was not improved by postnatal β adrenergic modifiers. Hindlimb‐specific glucose utilization rates in IUGR lambs were greater than normal across a range of insulin concentrations, but the proportion of glucose utilized by hindlimb tissues for oxidation was diminished. Moreover, primary hindlimb skeletal muscle had lower *ex vivo* glucose oxidation rates under basal and insulin‐stimulated incubation conditions. These results demonstrate that insulin sensitivities for non‐oxidative and oxidative glucose metabolism differ in skeletal muscle of IUGR lambs similar to whole‐body glucose metabolism in the IUGR fetus (Limesand *et al*. 2007; Brown *et al*. 2015). For IUGR fetuses, this could be at least partially explained by enhanced insulin signalling pathways, as IUGR fetal muscle expresses greater insulin receptor β and less p85α (Thorn *et al*. 2009). In IUGR lambs, however, skeletal muscle insulin receptor β concentrations were not affected at 2 weeks (Camacho *et al*. 2017) or 1 month of age (present study). Moreover, normal skeletal muscle citrate synthase activity in IUGR lambs indicates that diminished oxidative metabolic capacity is not due to reduced mitochondrial density. Rather, it may be due to altered pyruvate metabolism or impaired mitochondrial oxidative phosphorylation, as suggested by previous studies (Brown *et al*. 2015; Kelly *et al*. 2017; Pendleton *et al*. 2019). Equivalent lactate output, nutrient quotients and skeletal muscle glycogen content among all of our lambs indicates that the lower fractional glucose oxidation was not offset by greater glycolytic rates and glucose storage. Hindlimb glucose and lactate fluxes in IUGR fetal sheep further indicate that faster glycolytic rates are not a component of IUGR skeletal muscle programming (Rozance *et al*. 2018). Thus, additional studies will be needed to characterize the mechanisms responsible for the programmed defects in skeletal muscle glucose metabolism demonstrated by this study.

### Cardiovascular response

The chief aim of measuring the cardiovascular response in the present study was to determine the *in vivo* functional presence of the orally administered ADRβ modifiers. ADRβ1 agonist dobutamine increased the heart rate in both control and IUGR group, albeit to a lesser extent, but was unable to produce any change in the heart rate of the lambs in IUGR‐AR group. This demonstrates the functional presence of orally administered ADRβ1 antagonist atenolol. Unexpectedly, the treatment of ADRβ modifiers increased the relative size of the heart, specifically the left ventricle, which may indicate that the hypertrophy was from direct adrenergic regulation or from indirect adrenergic alteration of systemic blood pressures. The direct adrenergic mechanism may be in response to ADRβ2 activation and ADRβ1 inhibition to lower cardiomyocyte apoptosis via phosphoinositide 3 kinase and AKT pathways (Communal *et al*. 1999; Chesley *et al*. 2000; Gu *et al*. 2000; Zaugg *et al*. 2000; Zhu *et al*. 2001). In humans, IUGR is associated with greater risk for cardiovascular pathologies including hypertension, tachycardia and irregular cardiac growth (Brodszki *et al*. 2005; Crispi *et al*. 2010; Zanardo *et al*. 2011; Spence *et al*. 2012; Bjarnegard *et al*. 2013; Gaillard *et al*. 2013; Chatmethakul & Roghair, 2019). From this experiment, we show that heart rates were faster in IUGR lambs irrespective of the postnatal treatment and that the hearts from IUGR lambs were less responsive to the ADRβ1 agonist dobutamine. These findings warrant further investigation of ADRβ1 responsiveness and other factors regulating heart rate.

### Conclusion

Present findings allow us to conclude that metabolic pathologies in offspring that were born with IUGR manifest very early after birth and are the product of adaptive programming involving multiple tissues. Enhanced insulin secretion responsiveness to glucose is present in IUGR lambs at 1 week of age but subsides by 4 weeks of age, at which time β‐cell dysfunction is apparent. Whole‐body insulin sensitivity, which is also greater in the near‐term IUGR fetus and lamb at 2 weeks of age, had normalized by 1 month of age. However, disparities in skeletal muscle‐specific glucose metabolism persisted in lambs with IUGR. These include higher hindlimb glucose utilization rates that may be required to compensate for oxidative deficiencies of glucose in primary IUGR myocytes, thus causing fractional glucose oxidation rates to be lower in the hindlimb. Although the inherent discrepancies between glucose uptake and oxidation in muscle were unresolved by lactate release and glycogen content, greater transamination of pyruvate to alanine has been described in muscle of the IUGR fetus, which, if persistent, may explain the deficiencies in carbohydrate metabolism (Chang *et al*. 2019). Alternatively, the myocyte deficiencies in insulin‐stimulated glucose oxidation indicate that other factors contribute to glucose metabolism *in vivo*. We postulated that disrupted β adrenergic signalling plays an underlying role in the development of metabolic defects based on previously observed chronic hypercatecholaminaemia in the IUGR fetus. However, daily oral supplementation of pharmaceuticals intended to target suspected β adrenergic changes resulted in only modest improvements in insulin‐sensitive glucose metabolism and did not improve deficits in islet GSIS or skeletal muscle glucose metabolism in IUGR lambs. Moreover, targeted β adrenergic modifications did not improve the increased basal heart rates observed in IUGR lambs. Together, this study provides new insights regarding β‐cell dysfunction and muscle‐specific adaptations that predispose neonates that were born with IUGR to later life metabolic dysfunction.

## Additional information

### Competing interests

No conflicts of interest, financial or otherwise, are declared by the authors.

### Author contributions

All work was conducted at the University of Arizona's Agricultural Research Centre. D.T.Y., L.E.C. and S.W.L. conceived and designed the study; D.T.Y., L.E.C., A.C.K., L.V.S., M.A.D., A.T.A., M.J.A., R.G., R.E.A., K.K.P., W.W.H. and S.W.L. were involved in the acquisition, analysis and interpretation of data; D.T.Y., L.E.C. and S.W.L. prepared figures; D.T.Y., L.E.C. and S.W.L. drafted the manuscript. All authors edited and revised the manuscript. All authors have approved the final version of the manuscript and agree to be accountable for all aspects of the work in ensuring that questions related to the accuracy or integrity of any part of the work are appropriately investigated and resolved. All persons designated as authors qualify for authorship, and all those who qualify for authorship are listed.

### Funding

The Bill and Melinda Gates Foundation Global Health Grant Number OPP1066912 funded this project (Principal Investigator, S. W. Limesand). This work was also supported by the National Institutes of Health (NIH) R01DK‐084842 (Principal Investigator, S. W. Limesand). D. T. Yates was supported by National Institute of Food and Agriculture Postdoctoral Fellowship Award No. 2012‐67012‐19855 (Principal Investigator, D. T. Yates) and T32 HL007249 (Principal Investigator, C. C. Gregorio). L. E. Camacho was supported by National Institute of Food and Agriculture Postdoctoral Fellowship Award No. 2015‐03545 (Principal Investigator, L. E. Camacho) and T32 HL007249 (Principal Investigator, C. C. Gregorio). A. T. Antolic was supported by T32 HL007249 (Principal Investigator, C. C. Gregorio). W. W. Hay was supported by NIH T32 HD007186 (Principal Investigator and Project Director), NIH K12 HD068372 (PD) and NIH UL1TR001082 (Component Director).
